# Prophylactic heparin and mortality in patients with acute respiratory failure in the ICU: A retrospective cohort study based on the MIMIC-IV database

**DOI:** 10.1097/MD.0000000000050678

**Published:** 2026-09-11

**Authors:** Saichan Xu, Wenchao Mao, Changqin Chen, Changyun Zhao, Minjia Wang

**Affiliations:** a Department of Critical Care Medicine, Quzhou Kecheng People’s Hospital, Quzhou, Zhejiang, China; b Department of Critical Care Medicine, Zhejiang Hospital, Hangzhou, Zhejiang, China.

**Keywords:** acute respiratory failure, heparin, MIMIC-IV, mortality, propensity score matching

## Abstract

This study evaluates the association between prophylactic heparin use and mortality in intensive care unit (ICU) patients with acute respiratory failure (ARF). A retrospective cohort study was conducted using the Medical Information Mart for Intensive Care IV database. Adult ICU patients diagnosed with ARF were included. Patients receiving subcutaneous unfractionated heparin (5000 units) within 72 hours of ICU admission were compared with non-recipients. Propensity score matching was used to balance baseline characteristics. The primary outcome was 28-day all-cause mortality; secondary outcomes included in-hospital mortality and length of stay. Of 880 eligible patients, 703 received heparin and 177 did not. After 1:1 propensity score matching, 256 patients were analyzed (128 per group). Heparin use was associated with significantly lower 28-day all-cause mortality (32.03% vs 52.34%, *P* = .002) and in-hospital mortality (28.12% vs 46.88%, *P* = .003). Multivariable Cox regression confirmed reduced mortality risks (28-day hazard ratio = 0.46, 95% confidence interval: 0.30–0.69; in-hospital hazard ratio = 0.45, 95% confidence interval: 0.29–0.70). Subgroup analyses consistently supported these findings. However, heparin use was associated with longer ICU and hospital length of stay. Early prophylactic heparin administration is independently associated with reduced short-term mortality in ICU patients with ARF, supporting its potential role in comprehensive ARF management.

## 1. Introduction

Acute respiratory failure (ARF) is one of the most common critical illnesses in the intensive care unit (ICU), accounting for approximately 30 to 60% of all ICU patients, and its short-term mortality rate can reach over 30 to 50%.^[1,2]^ ARF is often triggered by various causes such as lung infection, acute respiratory distress syndrome (ARDS), sepsis, or atelectasis after major surgery, and is manifested as hypoxia and/or hypercapnia, accompanied by systemic inflammatory response and coagulation disorder.^[3,4]^ Overactivation of the coagulation system not only exacerbates microthrombosis but also further damages the alveolar-capillary barrier through inflammation-coagulation crosstalk, forming a vicious cycle.^[5,6]^ Therefore, intervention targeting the coagulation system can theoretically reduce the microthrombus burden, improve pulmonary circulation perfusion, and reduce the risk of multiple organ dysfunction.

As a classic anticoagulant, heparin has multiple effects, including anti-inflammation, regulation of endothelial function, and inhibition of neutrophil chemotaxis, in addition to its antithrombotic effect.^[7,8]^ Randomized controlled trials and meta-analyses have suggested that low-dose prophylactic heparin can reduce 28-day mortality in patients with sepsis or ARDS.^[9–11]^ However, these studies have been limited by small sample sizes and have often focused on specific etiologies, such as COVID-19-related ARDS.^[12,13]^ To date, evidence is lacking on the impact of prophylactic heparin on mortality in an unselected ICU population with ARF. To fill this evidence gap, we leveraged the large-scale, real-world Medical Information Mart for Intensive Care IV (MIMIC-IV) database and rigorously evaluated the impact of prophylactic subcutaneous unfractionated heparin (UFH) on 28-day and in-hospital mortality among ICU patients with ARF. Propensity-score matching (PSM) was employed to minimize confounding and deliver robust evidence for clinical decision-making.

## 2. Materials and methods

### 2.1. Data source

This study utilized data from the MIMIC-IV database (version 3.0),^[14]^ a large, publicly accessible dataset containing more than 50,000 deidentified ICU patient records from Beth Israel Deaconess Medical Center (Boston, MA, USA), spanning from 2008 to 2022. After completing the Collaborative Institutional Training Initiative program, 1 author (Changyun Zhao) was granted access to the MIMIC-IV database (certification number: 65303679). The study was conducted in accordance with the Declaration of Helsinki. The requirement for informed consent was waived because only anonymized data were analyzed. Ethical approval was obtained from the institutional review board of Beth Israel Deaconess Medical Center. This manuscript follows the guidelines outlined in the Strengthening the Reporting of Observational Studies in Epidemiology (STROBE) statement.^[15]^

### 2.2. Study population

The study included patients who were over 18 years of age, admitted to the ICU for the first time, and diagnosed with ARF during their ICU stay. Patients were excluded if they were using other anticoagulants (rivaroxaban, warfarin, enoxaparin, argatroban, and fondaparinux), or if heparin was administered for non-prophylactic indications (e.g., anticoagulation during parenteral nutrition or pacemaker therapy) or at therapeutic doses. Patients with absolute contraindications to heparin were also excluded, including those with active bleeding, coagulation disorders, recent major surgery, or abnormal laboratory values (platelet count < 50 × 10^9^/L or international normalized ratio > 2.5). ARF^[16]^ is defined as a rapid-onset impairment (typically developing within minutes to hours) in the ability to maintain adequate gas exchange, meeting at least one of the following criteria: Hypoxemic Type (Type I): arterial partial pressure of oxygen (PaO_2_) < 60 mm Hg (measured at sea level without supplemental oxygen); Hypercapnic Type (Type II): arterial partial pressure of carbon dioxide (PaCO_2_) > 50 mm Hg with progressive acidemia (pH < 7.35).

### 2.3. Data collection

Data extraction was conducted using Structured Query Language scripts from a publicly available GitHub repository (https://github.com/MIT-LCP/mimic-iv). The extracted variables included demographic data (age, race, ethnicity, and sex) and vital signs on admission (heart rate; systolic, diastolic, and mean blood pressure; body temperature; respiratory rate; and oxygen saturation [Spo2]). Information on preexisting comorbidities was also collected, including chronic kidney disease, congestive heart failure, hypertension, diabetes, chronic obstructive pulmonary disease, cirrhosis, and cancer. Laboratory parameters collected within 24 hours of ICU admission included the white blood cell count, platelet count, glucose level, hemoglobin level, potassium, sodium, calcium, chloride, creatinine, phosphate, magnesium, bicarbonate, and total bilirubin levels. Severity scores were recorded using the Sequential Organ Failure Assessment (SOFA), Glasgow Coma Scale, and Acute Physiology and Chronic Health Evaluation III (APACHE III). Treatment-related variables included renal support with continuous renal replacement therapy (CRPT), pharmacologic interventions such as vasopressors, and mechanical ventilation.

### 2.4. Exposure and outcomes

The exposure factor of interest was prophylactic heparin use during ICU hospitalization, administered subcutaneously at 5000 units per dose within 72 hours after ICU admission. Data on heparin administration were obtained from electronic prescription records, and patients with incomplete or unclear administration records were excluded from the analysis. The primary outcome was 28-day all-cause mortality, defined as death of any cause within 28 days of ICU admission. The secondary outcomes were in-hospital mortality (death during hospitalization), ICU length of stay (LOS; measured in days from ICU admission to discharge or death), and hospital LOS (measured in days from hospital admission to discharge or death).

### 2.5. Statistical analysis

Patients were stratified into 2 groups based on heparin exposure: the heparin group and the no-heparin group. Normally distributed continuous variables were reported as mean ± standard deviation and compared using analysis of variance. Non-normally distributed continuous variables were expressed as median (interquartile range) and analyzed using the Kruskal–Wallis test. Categorical variables were summarized as frequency (%) and compared using the chi-square test. To address baseline imbalances, 1:1 nearest-neighbor PSM was performed with a caliper width of 0.05 logits of the standard difference.^[17]^ Covariates included in the PSM model were age, sex, race, vital signs, laboratory results, comorbidities, severity scores (e.g., SOFA, APACHE III, and Glasgow Coma Scale), and treatments. Balance between groups was assessed using the standardized mean difference (SMD), with an SMD of < 0.10 indicating adequate balance. Cox proportional hazards regression was used to evaluate the association between heparin exposure and both 28-day and in-hospital mortality. The Mann–Whitney *U* test was used to compare ICU and hospital LOS between groups. Kaplan–Meier survival curves and log-rank tests were applied to analyze 28-day survival outcomes. Prespecified subgroups (e.g., age, sex, and comorbidities) were examined to assess the robustness of the findings. Variables with more than 20% missing data were excluded, while the remaining missing values were imputed using multiple imputation.^[18]^ All analyses were conducted using R software, version 3.3.2 (The R Foundation for Statistical Computing), and a two-tailed *P*-value of < .05 was considered statistically significant.

## 3. Results

### 3.1. Patient characteristics

**Figure 1.**
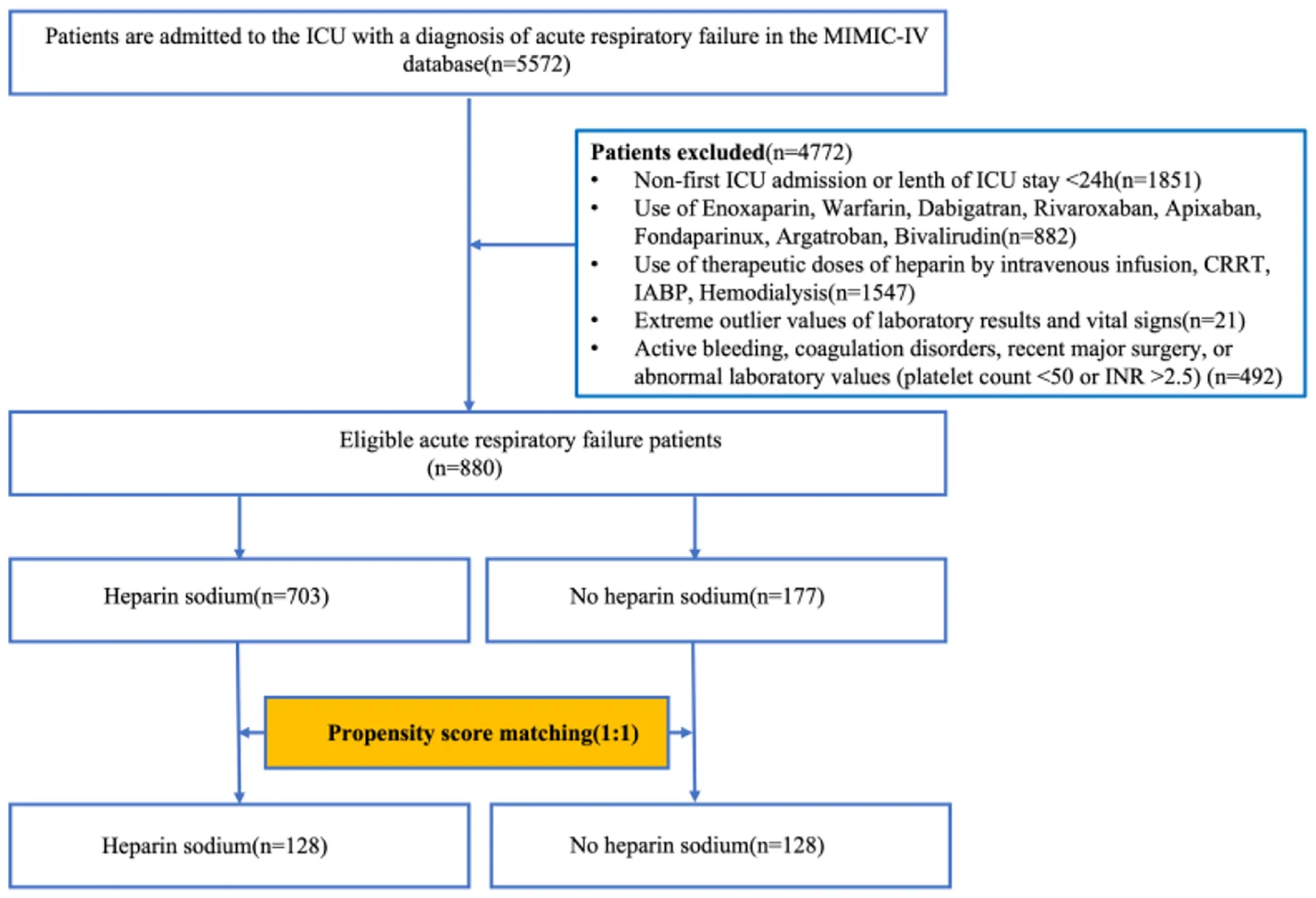
Flowchart of participant screening. CRRT = continuous renal replacement therapy, ICU = intensive care unit, MIMIC-IV = Medical Information Mart for Intensive Care IV.

Figure 1 presents the flowchart of participant screening. From the MIMIC-IV database, 5572 patients were admitted to the ICU with a diagnosis of sepsis-associated ARF. After excluding 4772 patients based on specific criteria (non-first ICU admission or length of ICU stay < 24 hours, use of certain anticoagulants, use of therapeutic heparin or specific procedures, and extreme outlier values), 880 eligible patients were included. Among them, 703 patients received heparin and 177 did not. After PSM (1:1), 128 patients were included in each group, resulting in a total matched cohort of 256 patients.

Table 1 presents the comparison of baseline characteristics and clinical indicators between heparin nonusers and users before and after PSM. Prior to matching, significant differences were observed between the non-heparin (n = 177) and heparin groups (n = 703). Heparin users had a higher proportion of females (48.79% vs 43.50%, SMD = 0.106). Clinically, heparin users exhibited markedly lower rates of cirrhosis (1.85% vs 9.04%; SMD = 0.321, *P* < .001) and cancer (11.38% vs 19.77%; SMD = 0.233, *P* = .005). Laboratory analyses revealed heparin users had higher platelet counts (median 224.00 vs 213.00 × 10^9^/L; SMD = 0.091) and hemoglobin levels (11.14 vs 10.40 g/dL; SMD = 0.323, *P* < .001), yet lower lactic acid (1.90 vs 2.12 mmol/L; SMD = 0.242, *P* = .013). Treatment differences included lower CRRT utilization (0.85% vs 2.82%; SMD = 0.147) and comparable vasopressor use (33.00% vs 34.46%; SMD = 0.031) among heparin users. Severity scores further showed lower APACHE III (48 [37–63] vs 54 [39–75]; SMD = 0.286, *P* = .005) and SOFA scores (6 [3–8] vs 6 [4–9]; SMD = 0.196, *P* = .033) in this group.

**Table 1:** Baseline characteristics of patients in the full cohort and propensity score-matched cohort.

Variable	Before	PSM	SMD	*P*-value	After	PSM	SMD	*P*-value
	No heparin	Heparin			No heparin	Heparin		
	(N = 177)	(N = 703)			(N = 128)	(N = 128)		
Gender, n (%)			0.106	.24			0.015	1.000
F	77 (43.50)	343 (48.79)			59 (46.09)	58 (45.31)		
M	100 (56.50)	360 (51.21)			69 (53.91)	70 (54.69)		
Race group, n (%)				.785				.581
Asian	7 (3.95)	29 (4.13)	0.008		4 (3.13)	4 (3.13)	0	
Black	20 (11.30)	81 (11.52)	0.007		11 (8.59)	16 (12.50)	0.127	
Others	36 (20.34)	167 (23.76)	0.082		28 (21.88)	33 (25.78)	0.092	
White	114 (64.41)	426 (60.60)	0.079		85 (66.41)	75 (58.59)	0.162	
Age, Median (Q1, Q3)	70.00 (57.00–83.00)	67.00 (55.00–81.00)	0.072	.375	71.00 (58.50–83.00)	64.00 (52.50–77.00)	0.263	.029
Comorbidities, n (%)								
Chronic kidney disease, n (%)	21 (11.86)	119 (16.93)	0.145	.126	17 (13.28)	12 (9.38)	0.123	.430
Congestive heart failure, n (%)	62 (35.03)	232 (33.00)	0.043	.673	47 (36.72)	42 (32.81)	0.082	.600
Diabetes, n (%)	49 (27.68)	192 (27.31)	0.008	.996	38 (29.69)	36 (28.13)	0.034	.890
Cirrhosis, n (%)	16 (9.04)	13 (1.85)	0.321	<.001	2 (1.56)	4 (3.13)	0.103	.680
Cancer, n (%)	35 (19.77)	80 (11.38)	0.233	.005	14 (10.94)	16 (12.50)	0.049	.846
Cerebrovascular, n (%)	24 (13.56)	70 (9.96)	0.112	.211	18 (14.06)	13 (10.16)	0.120	.443
Laboratory parameters								
WBC (10^9^/L), Median (Q1, Q3)	12.50 (8.50–18.30)	11.50 (8.00–16.00)	0.205	.039	12.20 (8.55–18.15)	11.20 (7.90–16.65)	0.074	.335
Platelets (10^9^/L), Median (Q1, Q3)	213.00 (133.00–284.00)	224.00 (167.00–288.00)	0.091	.071	213.50 (140.50–280.00)	224.00 (165.50–275.00)	0.014	.587
Glucose (mg/dL), Median (Q1, Q3)	135.00 (104.00–176.00)	132.00 (108.00–181.00)	0.002	.802	147.00 (109.50–194.50)	128.50 (102.00–192.00)	0.195	.169
Calcium (mg/dL), Median (Q1, Q3)	8.10 (7.40–8.60)	8.20 (7.60–8.70)	0.141	.042	8.10 (7.40–8.60)	8.11 (7.55–8.70)	0.061	.544
Hemoglobin (g/dL), Median (Q1, Q3)	10.40 (9.30–11.80)	11.14 (9.70–12.50)	0.323	<.001	10.80 (9.50–12.15)	11.20 (9.80–12.25)	0.171	.132
Creatinine (mg/dL), Median (Q1, Q3)	1.00 (0.70–1.60)	1.00 (0.70–1.50)	0.098	.456	1.05 (0.80–1.75)	1.10 (0.70–1.46)	0.289	.296
TBIL (mg/dL), Median (Q1, Q3)	1.00 (0.50–1.00)	1.00 (0.50–1.00)	0.147	.814	0.85 (0.50–1.00)	1.00 (0.45–1.00)	0.256	.254
Lactic acid (mmol/L), Median (Q1, Q3)	2.12 (1.30–2.50)	1.90 (1.20–2.12)	0.242	.013	2.10 (1.30–2.30)	1.95 (1.20–2.30)	0.001	.494
Vital signs								
Temp (°C), Median (Q1, Q3)	37.56 (37.06–38.06)	37.56 (37.11–38.17)	0.099	.158	37.61 (37.06–38.17)	37.50 (37.17–38.03)	0.035	.574
Hr (bpm), Median (Q1, Q3)	111.00 (92.00–125.00)	106.00 (93.00–120.00)	0.169	.038	108.50 (91.00–125.00)	110.00 (97.00–121.00)	0.052	.609
Nbps (mm Hg), Median (Q1, Q3)	144.00 (128.00–155.00)	144.00 (130.00–161.00)	0.096	.296	144.00 (129.50–154.00)	141.00 (125.50–162.00)	0.082	.656
Nbpd (mm Hg), Median (Q1, Q3)	85.00 (74.00–94.00)	87.00 (76.00–98.00)	0.143	.112	86.00 (74.00–95.00)	87.00 (72.00–98.50)	0.033	.670
Nbpm (mm Hg), Median (Q1, Q3)	96.00 (88.00–106.00)	98.00 (87.00–109.00)	0.124	.234	97.50 (88.00–106.50)	99.30 (83.00–107.00)	0.024	.909
RR (insp/min), Median (Q1, Q3)	29.00 (24.00–33.00)	29.00 (25.00–33.00)	0.025	.524	29.00 (24.00–33.00)	28.00 (24.00–32.00)	0.182	.442
Treatments, n (%)								
CRRT, n (%)	5 (2.82)	6 (0.85)	0.147	.083	3 (2.34)	2 (1.56)	0.056	1.000
Ventilation, n (%)	125 (70.62)	490 (69.70)	0.02	.883	88 (68.75)	94 (73.44)	0.104	.491
Vasopressor, n (%)	61 (34.46)	232 (33.00)	0.031	.780	47 (36.72)	40 (31.25)	0.116	.429
Score								
APACHE III, Median (Q1, Q3)	54.00 (39.00–75.00)	48.00 (37.00–63.00)	0.286	.005	50.50 (37.00–71.00)	48.50 (36.00–67.50)	0.069	.703
GCS, Median (Q1, Q3)	15.00 (11.00–15.00)	15.00 (13.00–15.00)	0.148	.377	15.00 (12.00–15.00)	14.00 (10.00–15.00)	0.084	.273
SOFA, Median (Q1, Q3)	6.00 (4.00–9.00)	6.00 (3.00–8.00)	0.196	.033	6.00 (3.00–9.00)	6.00 (4.00–8.00)	0.023	.739

APACHE III = Acute Physiology and Chronic Health Evaluation III, CRRT = continuous renal replacement therapy, GCS = Glasgow Coma Sxcale, Hr = heart rate, Nbpd = noninvasive blood pressure diastolic, Nbpm = noninvasive blood pressure mean, Nbps = noninvasive blood pressure systolic, NMBAs = neuromuscular blocking agents, PSM = propensity score matching, Q1 = 25th percentile (first quartile), Q3 = 75th percentile (third quartile), RR = respiratory rate, SMD = standardized mean difference, SOFA = Sequential Organ Failure Assessment, Temp = temperature, WBC = white blood cell.

Following 1:1 PSM (128 matched pairs; total N = 256), all examined covariates achieved adequate balance, with the majority of SMD below 0.1 and nonsignificant *P*-values (*P* > .05), confirming the effectiveness of the PSM process. Although age showed a slight imbalance (SMD = 0.263, *P* = .029), the overall covariate balance was substantially improved compared to the pre-matching cohort.

### 3.2. Heparin and clinical outcomes

Table 2 compares clinical outcomes between non-heparin and heparin-treated ARF patients before and after PSM. Before matching (n = 880), heparin administration was associated with significantly lower 28-day all-cause mortality (33.85% vs 55.37%; *P* < .001) and in-hospital mortality (30.01% vs 50.28%; *P* < .001) compared to non-heparin treatment. Heparin-treated patients demonstrated longer median hospital LOS (7.13 [interquartile range 4.31–11.73] vs 4.87 [2.47–8.04] days; *P* < .001) and ICU LOS (3.91 [2.26–7.42] vs 2.49 [1.69–4.10] days; *P* < .001). After 1:1 PSM (n = 256, 128 per group), the mortality benefit persisted with heparin treatment, showing lower 28-day all-cause mortality (32.03% vs 52.34%; *P* = .002) and in-hospital mortality (28.12% vs 46.88%; *P* = .003). The prolonged resource utilization pattern remained significant for both hospital LOS (7.85 [4.23–12.03] vs 4.26 [2.38–7.69] days; *P* < .001) and ICU LOS (4.25 [2.51–8.16] vs 2.44 [1.68–4.10] days; *P* < .001).

**Table 2:** Outcomes in the 2 groups before and after PSM.

Outcome	Total	No heparin	Heparin	p_value
Before PSM				
28-d mortality	336 (38.18%)	98 (55.37%)	238 (33.85%)	<.001
In-hospital mortality	300 (34.09%)	89 (50.28%)	211 (30.01%)	<.001
Hospital LOS (d)	6.81 [3.77–10.90]	4.87 [2.47–8.04]	7.13 [4.31–11.73]	<.001
ICU LOS (d)	3.60 [2.04–6.82]	2.49 [1.69–4.10]	3.91 [2.26–7.42]	<.001
After PSM				
28-d mortality	108 (42.19%)	67 (52.34%)	41 (32.03%)	.002
In-hospital mortality	96 (37.5%)	60 (46.88%)	36 (28.12%)	.003
Hospital LOS (days)	6.02 [3.35–9.94]	4.26 [2.38–7.69]	7.85 [4.23–12.03]	<.001
ICU LOS (days)	3.28 [1.94–5.96]	2.44 [1.68–4.10]	4.25 [2.51–8.16]	<.001

ICU = intensive care unit, LOS = length of stay, PSM = propensity score matching.

**Figure 2.**
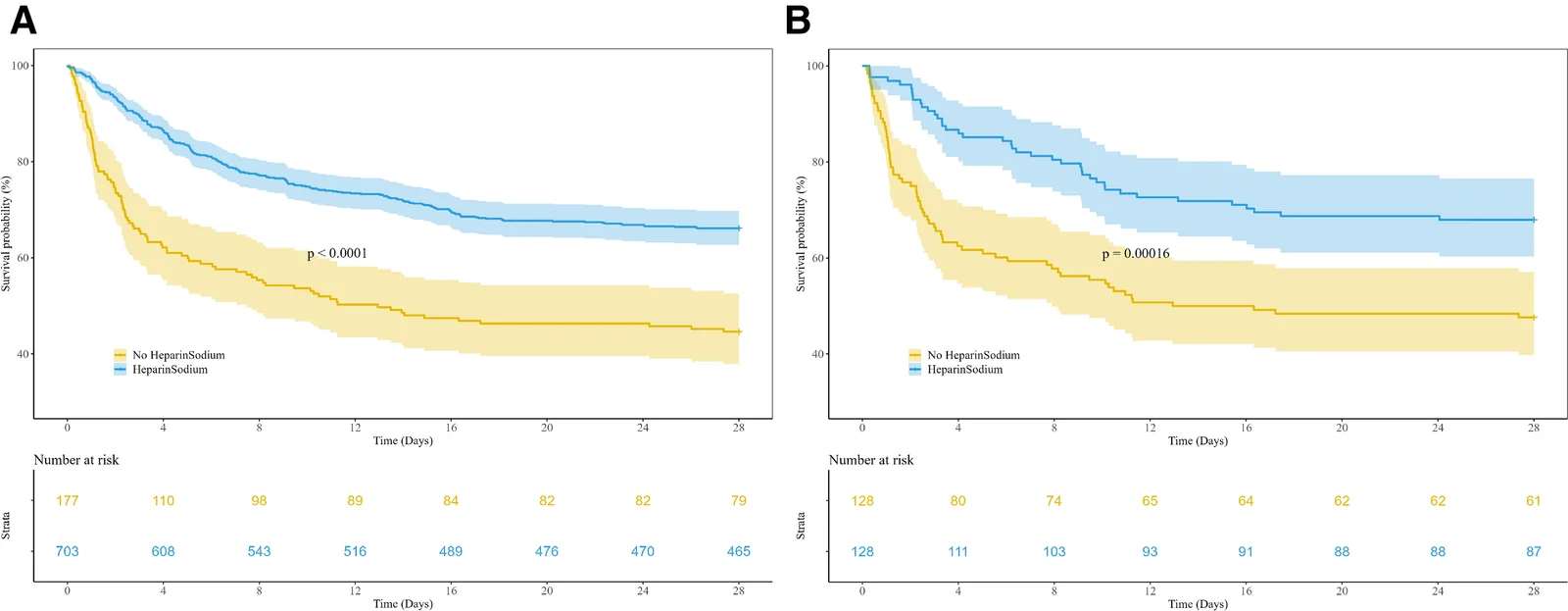
Kaplan–Meier survival curves between the heparin and non-heparin groups before and after propensity score matching. (A) Before propensity score matching. (B) After propensity score matching.

Figure 2 shows the Kaplan–Meier curves for 28-day survival in ARF patients before and after PSM. Before matching (703 heparin users vs 177 nonusers), heparin use was associated with significantly higher survival probability (log-rank *P* < .0001). After matching (128 patients per group), the survival benefit remained significant (log-rank *P* < .0001). The risk tables below the curves display the number of patients at risk at each time point, demonstrating consistent separation of survival curves throughout the 28-day follow-up period.

Table 3 displays the results of multivariate Cox regression analysis assessing the association between heparin use and clinical outcomes before and after PSM. Before matching (n = 880), heparin administration was significantly associated with reduced 28-day mortality (hazard ratio [HR] = 0.54, 95% confidence interval [CI]: 0.42–0.69; *P* < .001) and lower in-hospital mortality (HR = 0.55, 95% CI: 0.42–0.71; *P* < .001). After PSM (n = 256), these protective effects remained statistically significant: heparin use was associated with a 54% reduction in 28-day mortality risk (HR = 0.46, 95% CI: 0.30–0.69; *P* < .001) and a 55% reduction in in-hospital mortality risk (HR = 0.45, 95% CI: 0.29–0.70; *P* < .001). The consistency of HRs across both analytic approaches demonstrates robust protective associations independent of baseline confounders.

**Table 3:** Association between heparin use and outcomes.

Outcome	HR	CI	*P* value
Before PSM			
28-d mortality	0.54	0.42–0.69	<.001
In-hospital mortality	0.55	0.42–0.71	<.001
After PSM			
28-d mortality	0.46	0.30–0.69	<.001
In-hospital mortality	0.45	0.29–0.70	<.001

CI = confidence interval, HR = hazard ratio, PSM = propensity score matching.

### 3.3. Subgroup analysis

**Figure 3.**
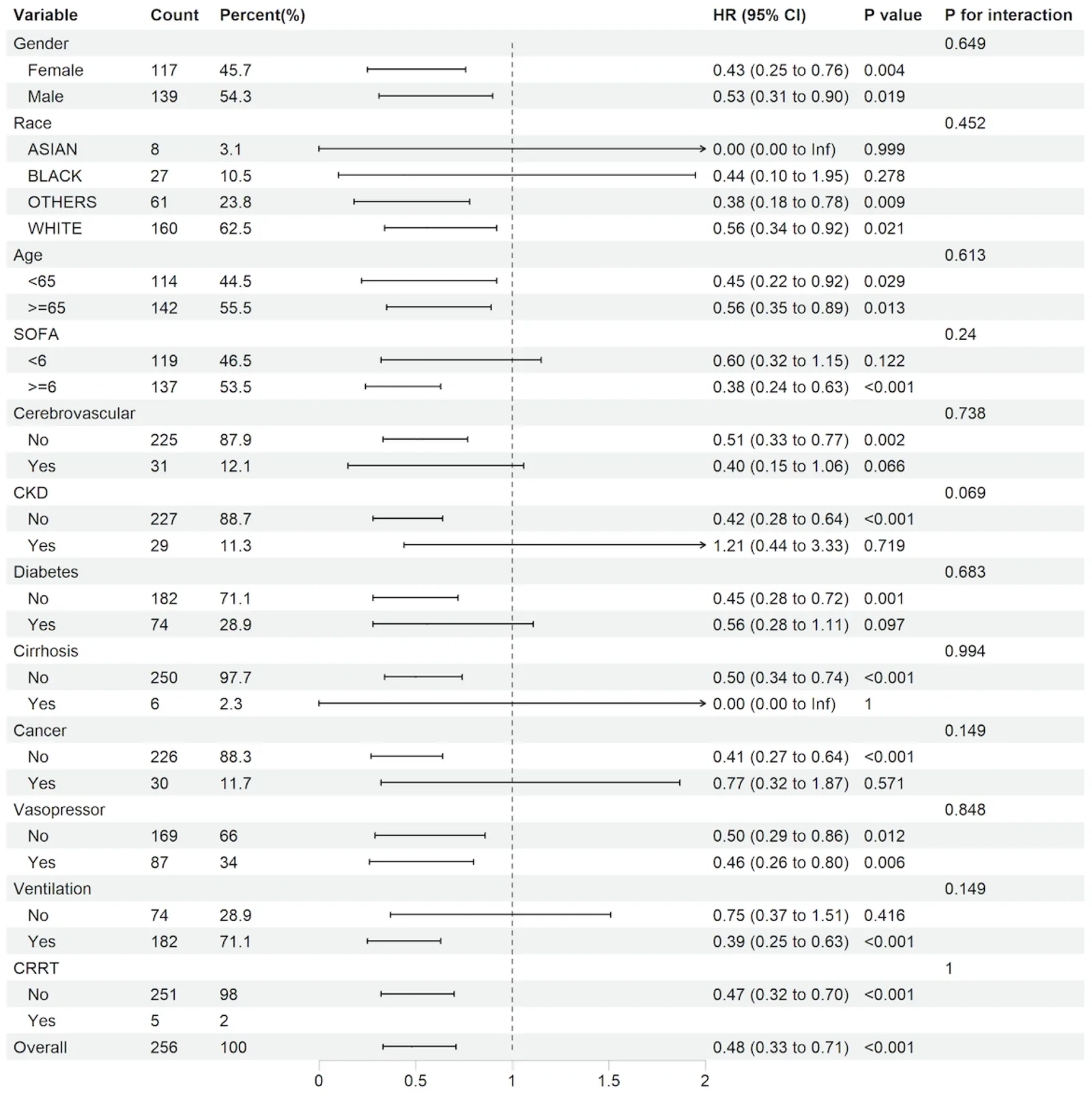
Subgroup analysis of the association between 28-day mortality and heparin treatment. CI = confidence interval, CKD = chronic kidney disease, CRRT = continuous renal replacement therapy, HR = hazard ratio, NMBAs = neuromuscular blocking agents, SOFA = Sequential Organ Failure Assessment.

Although an association was observed between heparin use and lower short-term mortality rates in patients with ARF, it remained unclear whether this correlation held across patients with varying clinical conditions. Therefore, a subgroup analysis was conducted on the matched cohort. The association between heparin treatment and 28-day mortality across different subgroups is illustrated in Figure 3. Among the 256 patients included in the analysis, heparin treatment was associated with a reduced 28-day all-cause mortality, with an overall HR of 0.48 (95% CI: 0.33–0.71), and a *P* value of < .001. Subgroup analyses revealed consistent protective effects of heparin across all predefined subgroups, including gender, race, age, SOFA score, comorbidities (cerebrovascular disease, chronic kidney disease, diabetes, cirrhosis, and cancer), and treatments (vasopressor, ventilation, and CRRT). All interaction *P*-values were nonsignificant (*P* for interaction > .05), indicating that the mortality reduction associated with heparin did not significantly differ across these clinical subgroups.

## 4. Discussion

This study, based on the MIMIC-IV database, conducted a retrospective cohort analysis to explore the association between prophylactic heparin use and mortality in ICU patients with ARF. The results consistently indicated that prophylactic heparin administration within 72 hours of ICU admission was significantly associated with reduced 28-day all-cause mortality and in-hospital mortality, even after PSM to balance baseline confounding factors. Meanwhile, heparin use was related to prolonged ICU and hospital lengths of stay, but this did not offset its protective effect on survival.

The significant reduction in mortality associated with prophylactic heparin observed in this study aligns with the findings of some previous related studies. A recent retrospective cohort study by Xiao et al based on the MIMIC-IV database further demonstrated that ARDS patients receiving prophylactic-dose heparin within 72 hours of ICU admission experienced a 29% reduction in in-hospital mortality risk, with this survival benefit persisting through 90 days.^[19]^ A large-scale cohort analysis by Smith et al, including 38,764 medically critically ill patients, showed that guideline-concordant pharmacologic thromboprophylaxis with either UFH or low-molecular-weight heparin (LMWH) reduced the risk of in-hospital death by approximately 10 % and significantly lowered pulmonary embolism-related mortality.^[20]^ In patients with ARF who require mechanical ventilation, prolonged immobility, endothelial injury, and systemic inflammation collectively generate a hypercoagulable state, predisposing to deep-vein thrombosis, pulmonary embolism, and ultimately microthrombosis-driven multiple organ dysfunction syndrome.^[21–23]^ Prophylactic heparin, by inhibiting the coagulation cascade, can effectively prevent the formation of these thrombotic events, thereby improving patient survival. The subgroup analysis in this study further confirmed the stability of this protective effect across different populations, including varying ages, genders, and comorbidity statuses, which enhances the credibility of the research results.

Although this study observed a significant association between heparin use and reduced mortality, its administration was also correlated with prolonged ICU and hospital LOS. This finding may reflect the complexity of heparin therapy: on the one hand, it improves organ perfusion and reduces the risk of multiple organ failure through anticoagulant and anti-inflammatory effects, thereby enhancing survival rates.^[24]^ It is also important to consider survivorship bias when interpreting the longer ICU and hospital lengths of stay observed in the heparin group. Patients who survive the acute phase of ARF inevitably accumulate more hospital days compared to those who die early, which may partially account for the prolonged LOS associated with heparin use in this study.

The findings of this study are consistent with several recent real-world studies. A 2024 retrospective cohort study of COVID-19 inpatients showed that anticoagulant therapy (including oral anticoagulants) was associated with reduced in-hospital mortality (adjusted odds ratio: 0.46, 95% CI: 0.24–0.87), with more pronounced benefits observed particularly in critically ill patients requiring mechanical ventilation.^[25]^ Another 2025 study based on the MIMIC-IV database found that low-dose aspirin (≤81 mg/d) reduced 28-day mortality risk by 55% (HR: 0.45, 95% CI: 0.29–0.7) in patients with sepsis-induced coagulopathy.^[26]^ These evidences collectively suggest that anticoagulant therapy may exert protective effects across critically ill patients with different etiologies by ameliorating hypercoagulable states and microcirculatory dysfunction.

The protective role of heparin in ARF patients may extend beyond its traditional anticoagulant mechanisms. In addition to preventing microthrombosis, heparin has been demonstrated to modulate immune function; for instance, LMWH treatment can improve immune markers such as CD3 + and CD4 + in septic patients, while reducing levels of inflammatory cytokines like interleukin-6 and tumor necrosis factor-α.^[27,28]^ These pleiotropic anti-inflammatory and immunomodulatory effects, potentially linked to the inhibition of neutrophil extracellular trap formation and endothelial function regulation, collectively contribute to its organ-protective actions.^[29–31]^

The results of this study hold significant clinical implications. For ARF patients in the ICU, early initiation (within 72 hours of ICU admission) of prophylactic-dose heparin may represent an effective strategy to improve short-term survival, particularly in the absence of large-scale randomized controlled trials targeting heterogeneous ARF populations. As an affordable and highly accessible medication, the potential survival benefits of heparin, if validated in broader populations, could yield substantial public health benefits.

However, this study has several limitations. First, as a retrospective observational study, despite employing PSM and multivariate Cox regression to control for confounders, the influence of unmeasured confounding factors (such as physician prescribing preferences, unrecorded bleeding risks, etc) cannot be entirely ruled out. Second, the data originate from a single center; although the MIMIC-IV database is extensive, generalizability requires further validation across populations from different regions and healthcare systems. Third, our definition of heparin exposure was based on electronic prescription records, precluding absolute certainty that medications were administered exactly as scheduled. Fourth, due to the lack of detailed documentation in the MIMIC‑IV database, we were unable to assess the incidence of bleeding complications – a critical safety outcome when evaluating heparin use in critically ill patients. Given the delicate balance between thrombosis and hemorrhage in ICU settings, the observed survival benefit must be interpreted with caution. Future studies should systematically capture bleeding events to enable a comprehensive risk‑benefit assessment of prophylactic heparin in ARF patients. Finally, due to missing relevant information in the database, we were unable to explore potential efficacy differences between LMWH and UFH, while existing evidence suggests possible variations in the effectiveness and safety of thromboprophylaxis between the two.

## 5. Conclusion

Analysis based on large-scale real-world data indicates that early use of prophylactic-dose heparin is independently associated with significantly reduced 28-day all-cause mortality and in-hospital mortality in ICU patients with ARF. This protective effect remained consistent across subgroups with different clinical characteristics. Although heparin use was associated with prolonged hospital stays, its survival benefits support considering its integration into the comprehensive management strategy for ARF patients without contraindications. This study provides new observational evidence for the use of heparin in ARF, warranting further validation through prospective research.

## Author contributions

**Conceptualization:** Saichan Xu, Minjia Wang.

**Data curation:** Saichan Xu, Wenchao Mao, Changqin Chen, Changyun Zhao.

**Formal analysis:** Wenchao Mao, Minjia Wang.

**Funding acquisition:** Changqin Chen.

**Investigation:** Changqin Chen, Changyun Zhao.

**Methodology:** Changyun Zhao.

**Writing – original draft:** Saichan Xu, Wenchao Mao, Changyun Zhao.

**Writing – review & editing:** Minjia Wang.
